# Sestrin2 protects dendritic cells against endoplasmic reticulum stress-related apoptosis induced by high mobility group box-1 protein

**DOI:** 10.1038/s41419-020-2324-4

**Published:** 2020-02-18

**Authors:** Li-Xue Wang, Xiao-Mei Zhu, Yi-Nan Luo, Yao Wu, Ning Dong, Ya-lin Tong, Yong-Ming Yao

**Affiliations:** 1 Trauma Research Center, Fourth Medical Center of the Chinese PLA General Hospital, Beijing, 100048 PR China; 2 First Medical Center of the Chinese PLA General Hospital, Beijing, 100853 PR China; 3 Department of Burns and Plastic Surgery, 924th Hospital of Chinese PLA, Guilin, 541002 PR China; 4 State Key Laboratory of Kidney Disease, the Chinese PLA General Hospital, Beijing, 100853 PR China

**Keywords:** Apoptosis, Immune cell death

## Abstract

Sestrin2 (SESN2) is a highly evolutionary conserved protein and involved in different cellular responses to various stresses. However, the potential function of SESN2 in immune system remains unclear. The present study was designed to test whether dendritic cells (DCs) could express SESN2, and investigate the underlying molecular mechanism as well as its potential significance. Herein, we firstly reported that SESN2 was expressed in DCs after high mobility group box-1 protein (HMGB1) stimulation and the apoptosis of DCs was obviously increased when SESN2 gene silenced by siRNA. Cells undergone SESN2-knockdown promoted endoplasmic reticulum (ER) stress (ERS)-related cell death, markedly exacerbated ER disruption as well as the formation of dilated and aggregated structures, and they significantly aggravated the extent of ERS response. Conversely, overexpressing SESN2 DCs markedly decreased apoptotic rates and attenuated HMGB1-induced ER morphology fragment together with inhibition of ERS-related protein translation. Furthermore, sesn2^−/−^-deficient mice manifested increased DC apoptosis and aggravated ERS extent in septic model. These results indicate that SESN2 appears to be a potential regulator to inhibit apoptotic ERS signaling that exerts a protective effect on apoptosis of DCs in the setting of septic challenge.

## Introduction

Dendritic cells (DCs), the most important potent antigen-presenting cells, are unique in their capacity to prime naive T cells and initiate immune response linking the innate immunity system with adaptive immune response^1,2^. Several reports have demonstrated that the profound depletion in the number of DCs, mainly suffered from apoptosis, bears mostly liability in the pathogenesis of sepsis and inflammatory diseases, and is potentially associated with the fatal outcome in septic or severe trauma patients^3–7^. However, the molecular regulatory mechanisms underlying the functional statues of DCs are poorly known at present. Nowadays, increasing evidences highlight the significant impact of endoplasmic reticulum (ER) stress (ERS) on maintaining cellular stability^8^. Unfolded protein response (UPR), triggered by the accumulation of unfolded or misfolded proteins within the lumen of ER in ERS, determines the cell destiny by adjusting the balance between cell adaption to stress and cell death resulting from disorder in ER homeostasis^9,10^. Recent studies have documented that ERS has important significance in functional modulation of DCs^11,12^.

Our previous studies proved that the overwhelming ERS response in splenic DCs in severe thermal injuries could be the main cause to initiate excessive apoptosis and dysfunction of DCs, thereby resulting in immunosuppression and even death^13–15^. The pathophysiological mechanism is highly related to an important late-acting cytokine high mobility group box-1 protein (HMGB1), which is increased markedly in sepsis and plays an important role in provoking inflammation and immune responses. Moreover, our previous studies demonstrated that HMGB1-induced ERS-related apoptosis in splenic DCs and had a complicated mechanism in mediating immune function of DCs. However, no therapeutic target in ERS signaling pathways has been elucidated in the immunomodulation of DCs.

Sestrins (SESNs), a highly conserved protein family, can be induced by various stresses, including oxidative stress^16^, DNA damage^17^, and hypoxia^18^. It was reported that SESNs protected cells against various stimuli by regulating autophagy, ERS, metabolism, and apoptosis. As the unique protein in SESNs family, SESN2 was found to be upregulated by the activation of ERS signaling pathways and be an important protective factor on relieving cell injury and cell death^19–24^. We proposed that SESN2 might be one of the favorable factors in immune regulation of DCs under ERS in the setting of sepsis. The present study aimed to verify the protective effects of SESN2 on apoptosis of DCs under stimulation with HMGB1 and during sepsis and tried to identify the key molecules in ERS-related apoptosis signaling pathways.

## Results

### HMGB1-induced apoptosis of DC2.4 cells

**Fig. 1:**
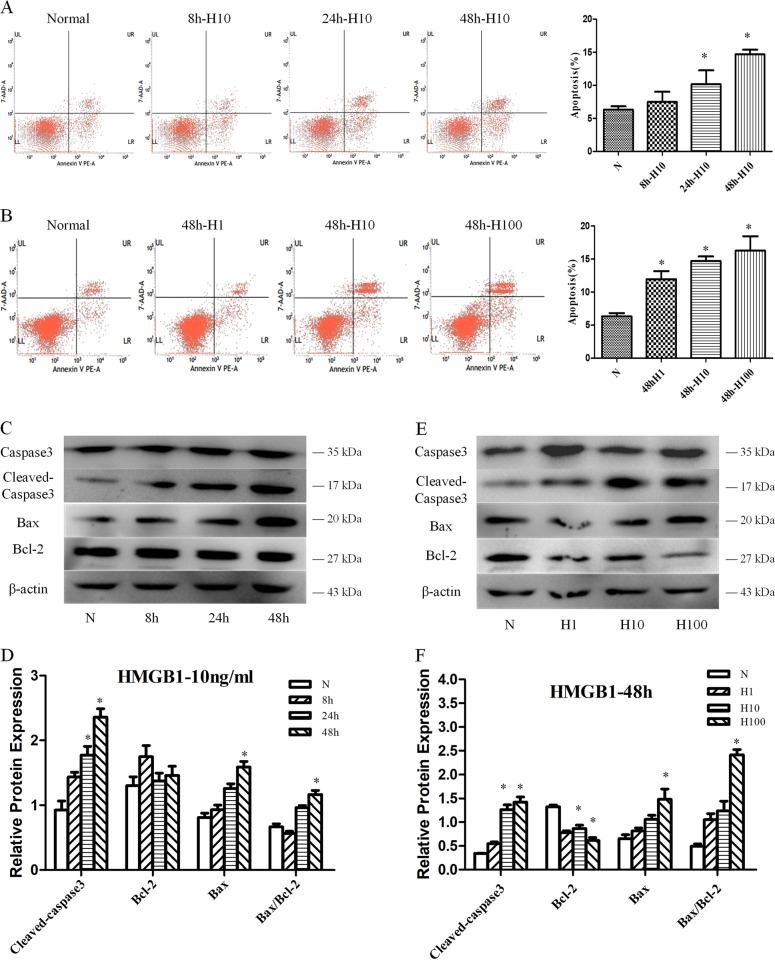
Treatment with HMGB1 enhanced cell apoptosis of DC2.4 cells. DC2.4 cells were cultured with HMGB1 (10 ng/ml) for different durations (0, 8, 24, and 48 h), or with various dosages of HMGB1 (1, 10, and 100 ng/ml) for 48 h. Cells cultured for 48 h without HMGB1 were used as the controls. **a**, **b** PE-Annexin V and 7-AAD were used to stain the treated DC2.4 cells and subjected to flow cytometry to assess cell apoptosis, *n* = 6 per group. **c**–**f** Caspase-3, cleaved-caspase-3, Bcl-2, and Bax expressions were measured as described in the section of methods, *n* = 3 per group. Data were presented as the mean ± SD. Statistical significance: ^*^P < 0.05 versus the control group.

DC2.4 cells were treated with various concentrations of HMGB1 for different intervals. As shown in Fig. 1a, b, HMGB1 stimulation in vitro could markedly increase the apoptotic rate of DC2.4 cells at dose of 1–100 ng/ml and cultured for 8–48 h, showing a time- and dose-dependent manner (all *P* < 0.05). We further examined level of caspase-3, cleaved-caspase-3, Bax, and Bcl-2 in DC2.4 cells stimulated with HMGB1. HMGB1 (especially at 10 and 100 ng/ml for 48 h) induced caspase-3 activation (cleaved), upregulated Bax protein, and suppressed Bcl-2 expression (Fig. 1c–f, *P* < 0.05). The Bax/Bcl-2 ratio was obviously elevated in HMGB1-stimulated groups when compared with the control group at 100 ng/ml for 48 h (Fig. 1c–f, *P* < 0.05).

### SESN2 was upregulated in DC2.4 cells with HMGB1-induced ERS

**Fig. 2:**
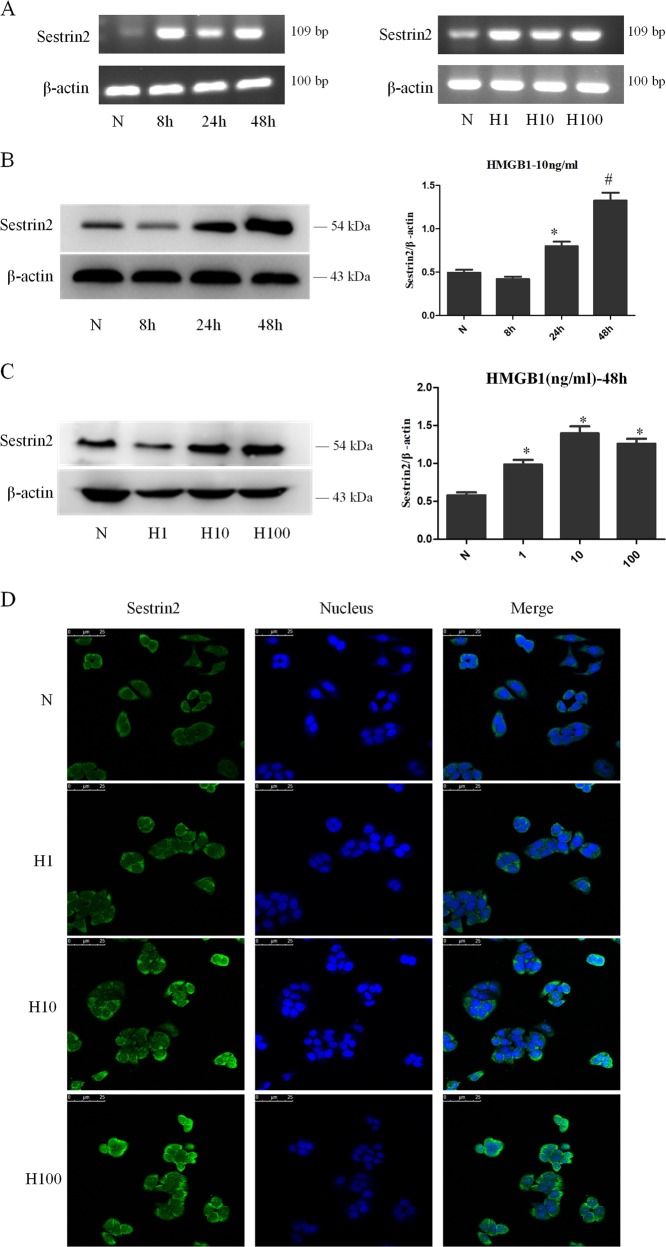
HMGB1 upregulated the expression of SESN2 in DC2.4 cells. **a** SESN2 mRNA expression were analyzed by PCR after DC2.4 cells stimulated with HMGB1 (10 ng/ml) for different intervals (0, 8, 24, and 48 h), or with various dosages of HMGB1 (1, 10, and 100 ng/ml) for 48 h. β-actin served as the internal standard. **b**, **c** Protein levels of SESN2 were analyzed by Western blotting after DC2.4 cells stimulated with 10 ng/ml HMGB1 for different time points or at various concentrations for 48 h. β-actin served as the internal standard, *n* = 3 per group. **d** Laser scanning confocal microscopy was employed to observe the expression of SESN2 protein in DC2.4 cells after HMGB1 treatment at various concentrations for 48 h, with FITC labeled SESN2 protein (green) and DAPI stained nucleus (blue) (×600), scale bar = 25 μm, *n* = 4 per group. Data were presented as mean ± SD of at least three independent experiments. Statistical significance: ^*^P < 0.05, ^#^*P* < 0.01 versus the control group.

To investigate the change of SESN2 expression in DCs after HMGB1 stimulation, DC2.4 cells were treated with HMGB1 at different concentrations for various intervals. As shown in Fig. 2, both mRNA and protein levels of SESN2 were upregulated in DC2.4 cells after HMGB1 stimulation. SESN2 mRNA expression in DC2.4 cells was significantly increased after treatment with HMGB1 for 24 h (Fig. 2a). Protein levels of SESN2 in DC2.4 cells were obviously upregulated when treated with 10 ng/ml HMGB1 for 48 h (Fig. 2b, c, *P* < 0.05). In addition, we used confocal laser scanning microscopy to further identify the expression of SESN2 protein in DC2.4 cells. Green fluorescence could be observed mainly in the cytoplasm of DC2.4 cells, and SESN2 expression was markedly enhanced after treatment with HMGB1 for 48 h at different concentrations (Fig. 2d).

### Protective effect of SESN2 on DC2.4 cells under HMGB1 stimulation

**Fig. 3:**
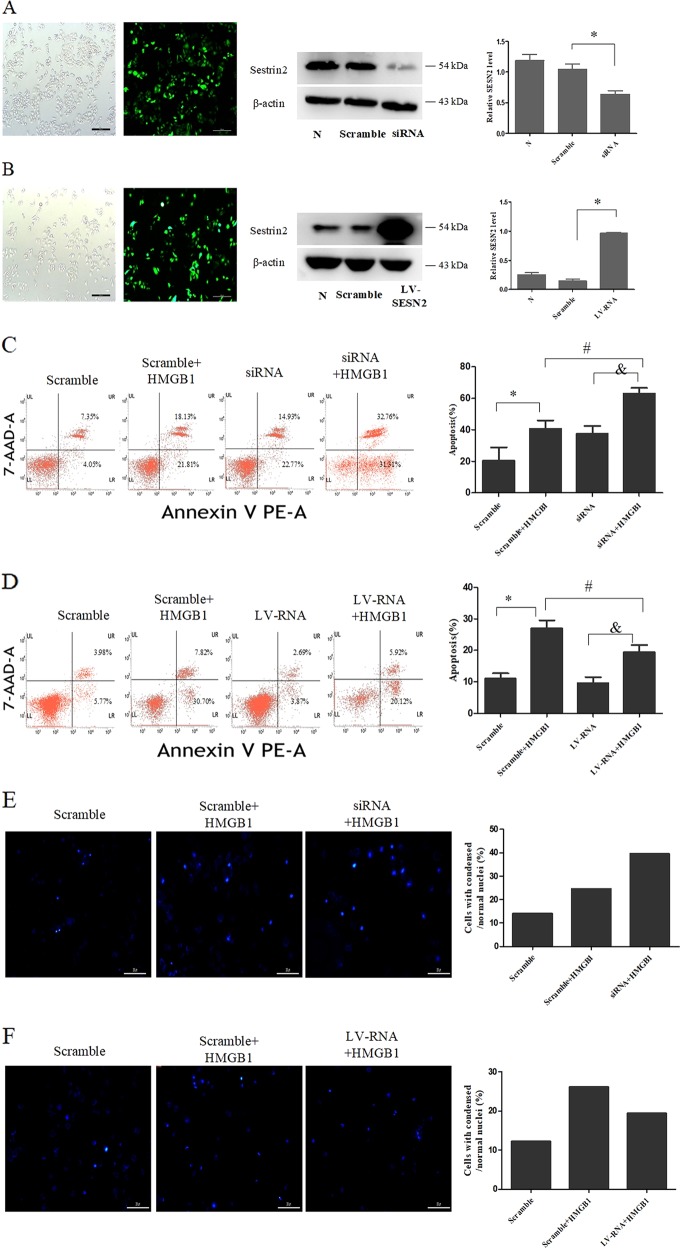

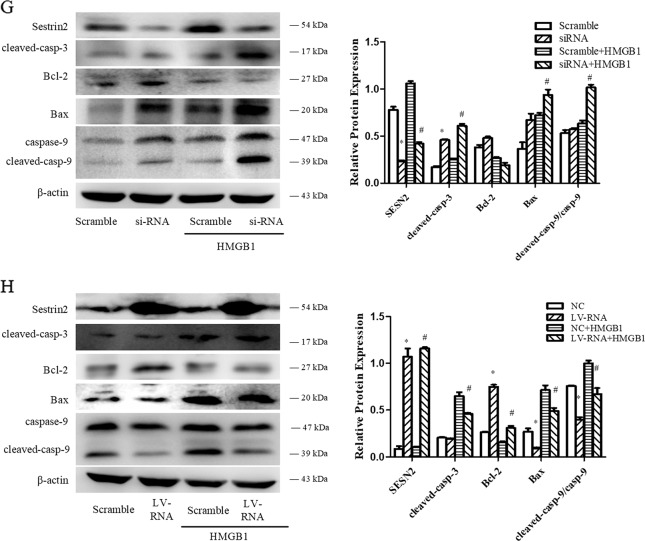
Protective effect of SESN2 on DC2.4 cells under HMGB1 stimulation. **a**, **b** Lentiviral vectors (SESN2 knockdown and SESN2 over-expression) were transduced DC2.4 cells, and scramble cells were transduced with blank vector. Transduced cells were analyzed by fluorescence microscopy (×400), scale bar = 200 μm, and then protein levels of SESN2 were determined by Western blotting. DC2.4 cells underwent SESN2-knockdown or SESN2-overexpression were incubated with 100 ng/ml HMGB1 for 48 h, and DC2.4 cells underwent blank vector treated with 100 ng/ml HMGB1 for 48 h served as the scramble group. Apoptotic rates of cells were measured with PE-Annexin-7-AAD by flow cytometry (**c**, **d**), and cell apoptosis was analyzed by Hoechst 33342 dye (blue) to observe nuclear condensation (×400, **e**, **f**), scale bar = 200 μm. Levels of SESN2, cleaved-caspase-3, cleaved-caspase-9, Bcl-2, and Bax were assessed by Western blotting (**g**, **h**), respectively. β-actin served as the internal standard. Values were represented as mean ± SD obtained from three independent experiments, *n* = 3 per group. Statistical significance: *P < 0.05 versus the scramble group; ^#^*P* < 0.05 versus the scramble group treated with HMGB1.

DC2.4 cells were transduced with recombinant lentiviruses that carried SESN2 siRNA (knockdown) or SESN2-LV-RNA (overexpression) (Fig. 3a, b). The results showed that protein expression of SESN2 was downregulated in the SESN2 siRNA group and dramatically upregulated in the SESN2 LV-RNA group, when compared with the scramble group (Fig. 3a, b).

To confirm the impact of SESN2 on cell apoptosis, cells were examined with Annexin-V-phycoerythrin (PE)/7-AAD quantification assay and Hoechst 33342 staining, respectively. As shown in Fig. 3c, the apoptotic rate of DC2.4 cells with SESN2-siRNA was obviously higher than the scramble group, especially under HMGB1 stimulation (Fig. 3c, *P* < 0.05). Hoechst 33342 staining also revealed more condensed or fragmented apoptotic nuclei in the SESN2-siRNA group than that in the scramble group (Fig. 3e). Furthermore, activation of caspase-3 and caspase-9 (critical factors of apoptosis) in the SESN2-siRNA group was markedly exacerbated when compared to the scramble group (Fig. 3g). Expression levels of the anti-apoptosis protein of Bcl-2 were decreased and the pro-apoptosis protein of Bax were increased after HMGB1 stimulation, and Bax/Bcl-2 ratio in the SESN2-siRNA group was significantly elevated when compared with the scramble group (Fig. 3g, *P* < 0.05).

On the contrary, the apoptotic rate of DC2.4 cells with SESN2-LV-RNA was effectively reduced in comparison to the scramble group after treatment with HMGB1 (Fig. 3d, f, *P* < 0.05). Levels of cleaved-caspase-3 and cleaved-caspase-9 were obviously decreased. The expression of antiapoptosis protein of Bcl-2 was upregulated, while Bax expression was downregulated, and Bax/Bcl-2 ratio in the SESN2-LV-RNA group was significantly decreased when compared with that in the scramble group (Fig. 3h, *P* < 0.05).

### Protective effect of SESN2 on DC2.4 cells was closely related to the inhibition of ERS-related apoptotic pathway

#### Co-localization of SESN2 and ER in DC2.4 cells

**Fig. 4:**
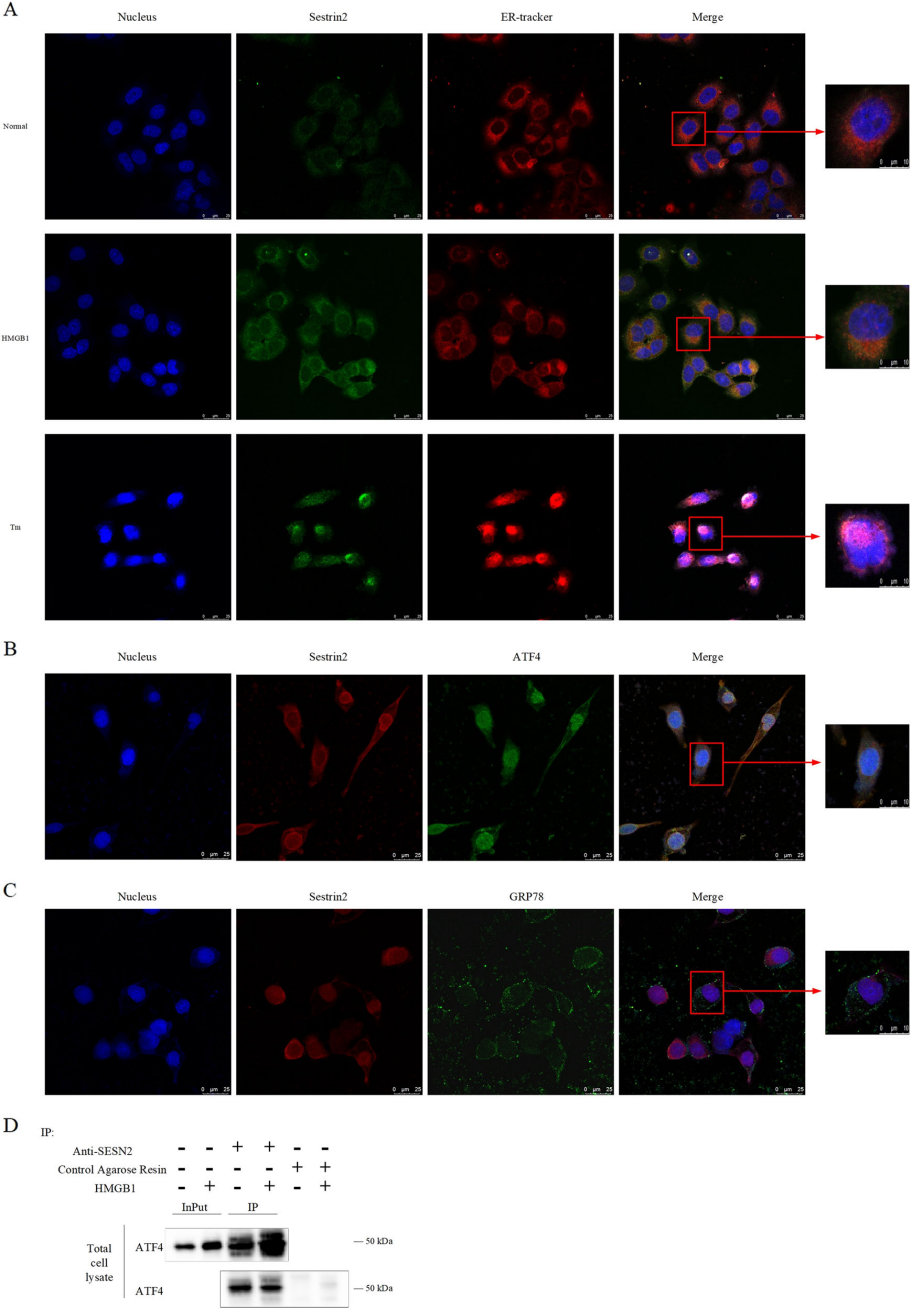
Co-localization of SESN2 and ER in DC2.4 cells (×600, ×1200). **a** Laser scanning confocal microscopy was used to observe SESN2 and ER in DC2.4 cells. It was shown by anti-SESN2 antibodies (green) and ER tracker (red) using indirect immunofluorescence and confocal microscopy. **b** It was observed by anti-SESN2 antibodies (red) and ATF4 (green) using indirect immunofluorescence and confocal microscopy. **c** It was shown by anti-SESN2 antibodies (red) and GRP78 (green) using immunofluorescence and confocal microscopy. **d** Immunoblot analysis of ATF4 in SESN2 was immunoprecipitated in total cell lysate. Negative control was using control agarose resin. Scale bar = 25 μm, scale bar = 10 μm, *n* = 3 per group. Its co-localization was shown in saffron yellow (DAPI stained nucleus, blue).

DC2.4 cells were stained with antibody to SESN2 (green) and ER-tracker probe (red). SESN2 was co-localized with the organelle of ER (Fig. 4a, shown in saffron yellow). The fluorescence intensity of SESN2 was enhanced after treatment with HMGB1 or tunicamycin (TM, prototypical ERS inducer). Meanwhile, result from co-localization using SESN2 antibody (red) and activating transcription factor (ATF)4 antibody (green) or glucose regulated protein (GRP)78 (green) showed that SESN2 could interact with ATF4 not GRP78 to regulate ERS (Fig. 4b, c, shown in saffron yellow). We further examined co-immunoprecipitation (co-IP) with SESN2 antibody and Western blot analysis showed SESN2 interacted with ATF4 in DC2.4 cells (Fig. 4d).

#### SESN2-protected DC2.4 cells against ERS-related apoptosis triggered by TM

**Fig. 5:**
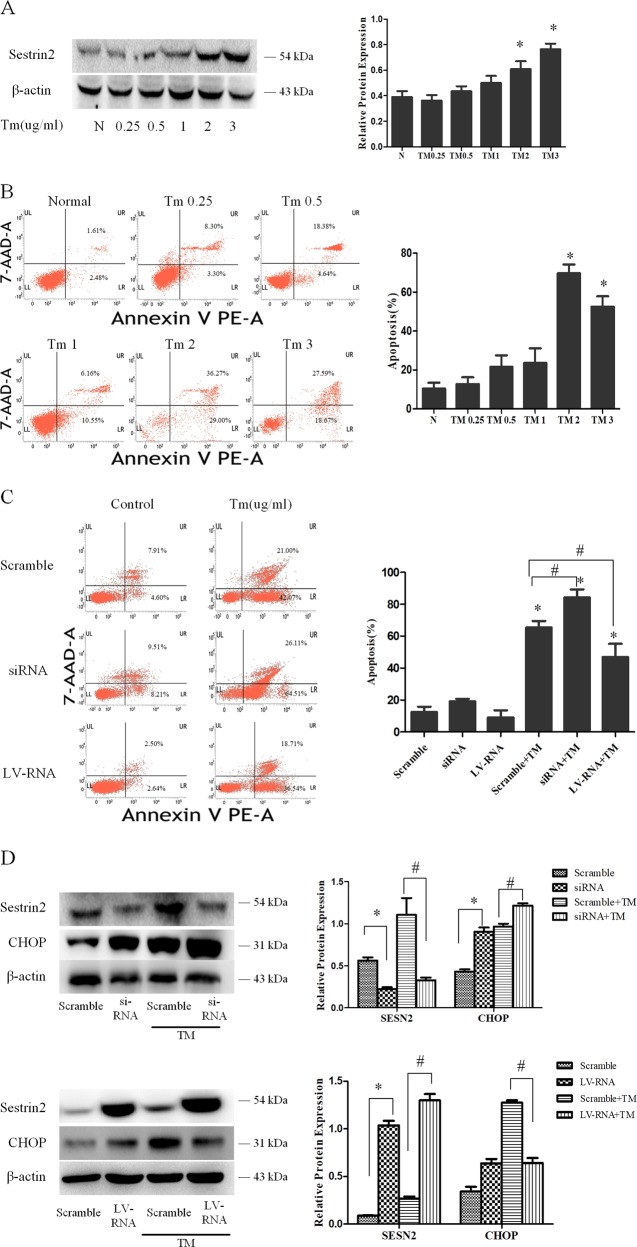
SESN2 protected DC2.4 cells against ERS-related apoptosis triggered by TM. DC2.4 cells were treated with various dosages of TM (0.25, 0.5, 1, and 100 ng/ml) for 48 h. Cells cultured for 48 h without TM were used as the controls. **a** PE-Annexin-V and 7-AAD were used to stain the treated DC2.4 cells and subjected to flow cytometry to assess cell apoptosis with different dosages of TM for 48 h. **b** SESN2 expression was determined by Western blotting. **c** After modulated SESN2 expression, PE-Annexin V and 7-AAD were used to stain the treated DC2.4 cells and flow cytometry was used to assess cell apoptosis after stimulated with 2 μg/ml TM for 48 h. **d** Expressions of SESN2 and CHOP were measured as described in the section of methods following treatment with 2 μg/ml TM for 48 h. β-actin served as the internal standard. Data of three independent experiments were presented as the mean ± SD. Statistical significance: ^*^P < 0.05 versus the scramble group; ^#^*P* < 0.05 versus the scramble group treated with HMGB1.

DC2.4 cells were treated with different concentrations of TM for 48 h, which resulted in significant apoptosis at 2 μg/ml (Fig. 5a, *P* < 0.05). Accordingly, SESN2 was markedly upregulated after TM stimulation (0.25–3 μg/ml for 48 h) in a dose-dependent manner (Fig. 5b). To investigate the protective effect of SESN2 on ERS-induced apoptosis, DC2.4 cells with SESN2 siRNA or SESN2-LV-RNA were treated with 2 μg/ml TM for 48 h. SESN2 gene silence resulted an increased apoptosis when compared with the scramble group (Fig. 5c, *P* < 0.05). Conversely, the apoptotic rate was decreased when SESN2 gene was overexpressed (Fig. 5c, *P* < 0.05). As an important component of the ERS-mediated apoptosis pathway, C/EBP homologous protein (CHOP) was obviously exacerbated after SESN2 knockdown and levels were lower in the SESN2 overexpression group in comparison with the scramble controls (Fig. 5d, *P* < 0.05), respectively.

#### HMGB1-induced apoptosis of DC2.4 cells through ERS-apoptotic pathway

**Fig. 6:**
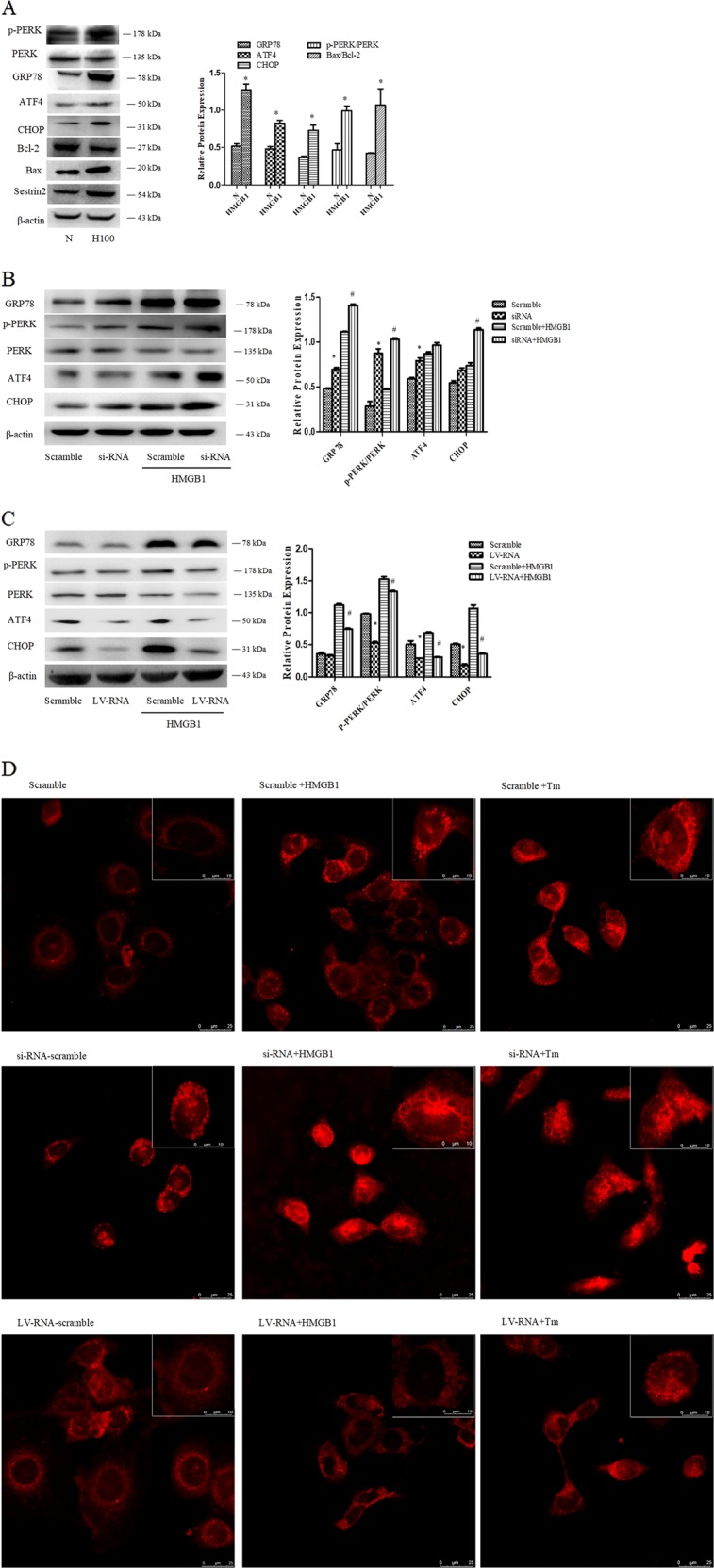
SESN2 protected DC2.4 cells against excessive ERS response induced by HMGB1. **a** Expressions of GRP78, p-PERK, PERK, ATF4, CHOP, Bcl-2, and Bax were determined by Western blotting after treatment with 100 ng/ml HMGB1 for 48 h. **b**, **c** Lentiviral vectors (SESN2 knockdown and SESN2 overexpression) were transduced DC2.4 cells, and scramble cells were transduced with blank vectors. After treated with 100 ng/ml HMGB1 for 48 h, expressions of GRP78, p-PERK, PERK, ATF4, and CHOP were determined by Western blot analysis. **d** The morphologic alteration of ER in each cell was evaluated by immunofluorescence staining and the extent of the ER morphologic change was compared with the scramble controls (×600, ×1200), scale bar = 25 μm, scale bar = 10 μm, *n* = 4 per group. ER was detected with ER-tracker red. β-actin served as the internal standard. Data were presented as the mean ± SD, *n* = 3 per group. Statistical significance: *P < 0.05 versus the control group.

As shown in Fig. 6a, treatment with 100 ng/ml HMGB1 for 48 h significantly increased expressions of ERS markers including GRP78 (*P* < 0.05), phosphorylation (p) of protein kinase RNA (PKR)-like ER kinase (PERK), PERK, and ATF4 (*P* < 0.05). Likely, CHOP was obviously increased following HMGB1 stimulation (*P* < 0.05). In addition, apoptotic-related protein Bax and Bcl-2 were analyzed, and the Bax/Bcl-2 ratio was increased in DC2.4 cells (*P* < 0.05). Thus, PERK-ATF4-CHOP-mediated cell death pathway might be activated in HMGB1-induced DC2.4 cell death.

#### SESN2-protected DC2.4 cells from excessive ERS response induced by HMGB1

ERS signaling pathways were examined in HMGB1-stimulated DC2.4 cells after SESN2-siRNA or SESN2-LV-RNA transfection. Silence of SESN2 markedly aggravated ERS response and enhanced ERS-related apoptosis. ERS markers including GRP78, p-PERK, and ATF4 were significantly upregulated. Protein level of CHOP, a pivotal component in ERS-related apoptotic pathway, also presented a substantial increase (Fig. 6b). By contrast, over-expression of SESN2 could inhibit ERS induced by HMGB1 with downregulation of ATF4 as well as CHOP and reduction of PERK phosphorylation (Fig. 6c, *P* < 0.05).

We further observed the changes of ER structure after interfering SESN2 expression. As shown in Fig. 6d, alteration and fragmentation of ER could be found in DC2.4 cells stimulated by HMGB1 or TM. Significantly fragmented and lumped changes were noticed in ER morphology of SESN2 silenced DC2.4 cells after treatment with 100 ng/ml HMGB1 or 2 μg/ml TM for 48 h. While SESN2-LV-RNA transfected DC2.4 cells maintained the structure of ER after HMGB1 or TM stimulation, almost the same interconnected networks as in the scramble group (Fig. 6d).

#### SESN2 inhibited PERK-CHOP pathway to protect DC2.4 cells against ERS-related cell apoptosis

**Fig. 7:**
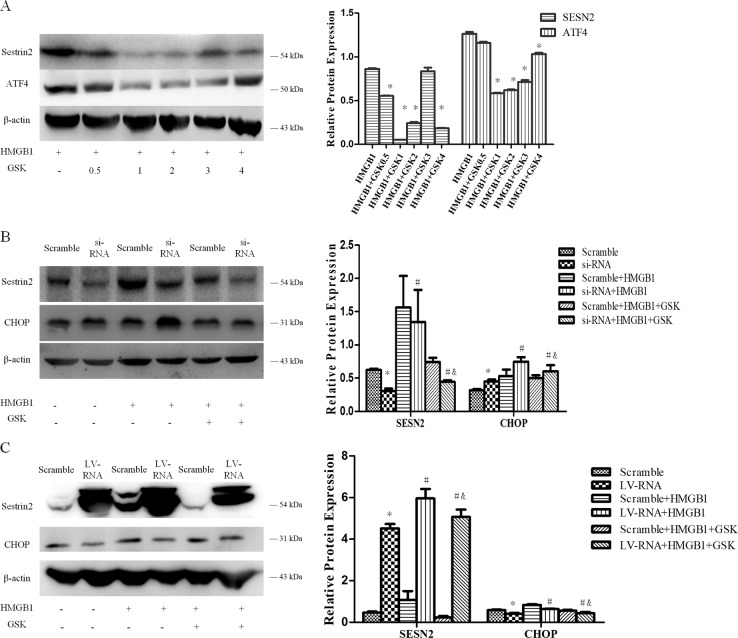
SESN2-inhibited PERK-CHOP pathway to protect DC2.4 cells from ERS-related cell apoptosis. **a** Various dosages of GSK2656157 was pretreated cells for 1 h, then stimulation with 100 ng/ml HMGB1 for 48 h. ATF4 and SESN2 in DC2.4 cells were determined by Western blotting. **b**, **c** After induction with siRNA or LV-RNA for 72 h, DC2.4 cells were treated with 100 ng/ml HMGB1 for 48 h, then SESN2 as well as ERS apoptotic-related protein CHOP were examined by Western blotting. β-actin served as the internal standard. Data were presented as the mean ± SD, *n* = 3 per group. Statistical significance: *P < 0.05 versus the scramble group; ^#^*P* < 0.05 versus the scramble group treated with HMGB1; ^&^*P* < 0.05 versus the scramble group treated with HMGB1 and GSK2656157.

To further investigate the potential signaling pathways with regard to protective impact of SESN2 on ERS-related cell apoptosis, DC2.4 cells were pretreated with various concentrations of PERK inhibitor (GSK2656157, 0.5–3 μmol/L) for 1 h, then exposed to 100 ng/ml HMGB1 for 48 h. Pretreatment with PERK inhibitor obviously attenuated ATF4 expression and reduced SESN2 induction by HMGB1, especially at 2 mmol/L of GSK2656157 (Fig. 7a). Blockade of PERK signaling pathway significantly decreased SESN2 and CHOP expressions, and diminished the protective actions of SESN2 in both SESN2 silenced and SESN2 overexpressed DC2.4 cells in comparison with the scramble controls (Fig. 7b, c).

### The protective impact of SESN2 on DCs in cecal ligation and puncture (CLP)-induced septic mice

**Fig. 8:**
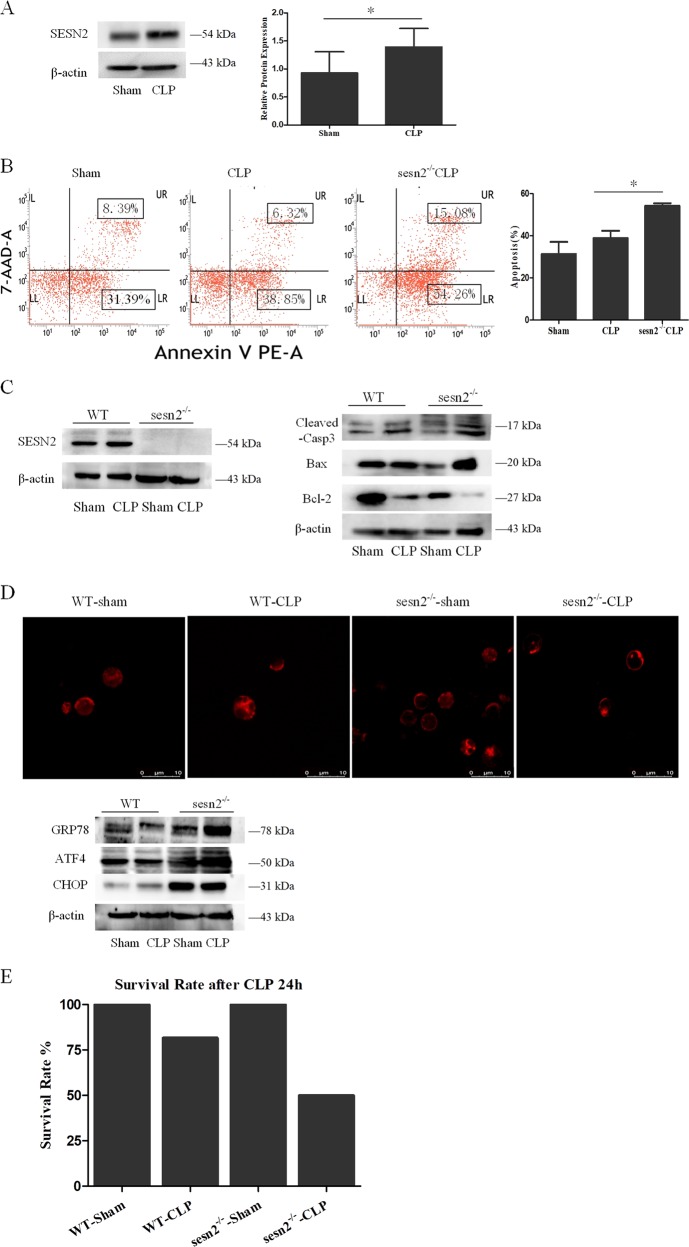
The protective effect of SESN2 on DCs in CLP-induced septic mice. Mice underwent a sham procedure or CLP. **a** SESN2 expression was determined by Western blotting at 24 h after after CLP. **b** PE-Annexin-V and 7-AAD were used to stain DCs and subjected to flow cytometry to assess cell apoptosis at 24 h after CLP in vivo. **c** Expressions of cleaved-caspase-3, Bcl-2, and Bax were measured as described in the section of methods after CLP procedure. **d** In the sesn2^−/−^ CLP group, the morphologic alteration of ER in each cell was evaluated by immunofluorescence staining and the extent of the ER morphologic change was compared with the WT CLP group (×1200). ER was detected with ER-tracker red. Levels of GRP78, ATF4, and CHOP were assessed by Western blotting. β-actin served as the internal standard. **e** The 24-h survival rate of mice in the sesn2^−/−^ CLP group was markedly lower than that in the WT CLP group. Data of three independent experiments were presented as the mean ± SD, *n* = 6 per sham group, *n* = 11–13 per CLP group. Statistical significance: *P < 0.05 for in vivo comparison of the sesn2^−/−^ CLP group versus the WT CLP group.

To study the pathophysiological role of SESN2 in vivo, we evaluated the protective effect of SESN2 on splenic DCs in a CLP-induced septic murine model. As shown in Fig. 8a, SESN2 was upregulated in splenic DCs from septic mice at 24 h. Knockout of SESN2 resulted in a higher apoptotic rate of DCs after CLP procedure, when compared with wild-type (WT) mice (Fig. 8b, *P* < 0.05). Accordingly, increases in proapoptotic proteins (BAX and cleaved-caspase-3) and decreases in antiapoptotic protein (Bcl-2) in CLP mice were reinforced in sesn2^−/−^ mice (Fig. 8c).

To further investigate the influence of SESN2 on ERS in DCs in CLP mice, we observed the morphologic changes of ER and the expression of ER chaperones. As shown in Fig. 8d, more extensive fragmented and lumped ER could be found in sesn2^−/−^ DCs, and protein levels of ATF4 and CHOP were also upregulated (Fig. 8d). Moreover, SESN2 deficiency strongly aggravated ERS responses in DCs during sepsis, as evidenced by upregulation of GRP78 and ATF4 in CLP mice (Fig. 8d). In addition, we compared the 24-h survival rate of CLP mice between sesn2^−/−^ mice and WT mice. These results showed that sesn2^−/−^ knockout aggravated CLP-induced mortality at 24 h (Fig. 8e).

## Discussion

Sepsis was now defined as life-threatening organ dysfunction caused by dysregulated host response to infection^25^. It is well documented that extensive apoptosis of splenic leukocytes, including T lymphocytes, macrophages, and DCs, occurs in sepsis and endotoxic shock, may contribute to the immunosuppressive characteristic of these disorders^26,27^. DCs are critical potent antigen-presenting cells that are key factors in immune response by initiating T lymphocytes. Previous studies indicated that the decreased number of splenic DCs in mice suffered from sepsis and blood DCs in septic patients played important roles in sepsis-induced immune paralysis^28,29^. The loss of DCs during sepsis appears to be a universal phenomenon, and it acts as a mark of immune depression. Therefore, it is entirely reasonable for us to believe that reduction and prevention of the apoptosis of DCs might improve and reverse the immunosuppressive state in the setting of sepsis. HMGB1, one of the key damage-associated molecular patterns, is an important late-acting inflammatory cytokine of sepsis that can be actively secreted by immune cells or passively released by injured or necrotic cells. It has been documented that HMGB1 is critically involved in host immune dysfunction and various diseases^30–32^, and it acts as a potent mediator of DC activation, maturation as well as survival^13–15,33–35^. In the present study, we investigated the proapoptotic effect of HMGB1 stimulation on DC2.4 cells in vitro, showing a time-dependent and dose-dependent manner. It is well-known that both antiapoptotic protein of Bcl-2 and proapoptotic protein of Bax are two members of Bcl-2 family, and Bax can activate the downstream signal caspase-3 promoting apoptosis^36^. Our results revealed that treatment with HMGB1 (10–100 ng/ml for 48 h) markedly induced activation of caspase-3, upregulation of Bax, and downregulation of Bcl-2.

Recently, SESN2 is reportedly involved in many cellular responses to various stresses. In our study, stimulation with HMGB1 for 48 h could obviously induce the upregulation of SESN2 in DC2.4 cells in a dose-dependent pattern in vitro, and the expression of SESN2 was enhanced at 24 h after CLP in vivo. Many experiments have proved that SESN2 exerts cytoprotective function and prevents cell apoptosis via different mechanisms. For instance, Ben-Sahra et al.^37^ found that SESN2 protected cells from energetic stress-mediated apoptosis by maintaining the energy homeostasis. Perk et al.^20^ showed that SESN2-knockdown hepatocytes were hypersusceptible to obesity-induced apoptosis. A recent study suggested that SESN2 could counteract Toll-like receptor (TLR)-mediated proinflammatory signaling and cell apoptosis in macrophages^38^. Consistent with these observations, we noted that knockdown of SESN2 increased cell apoptosis, enhanced the activation of capsae-3, caspase-9, and elevated the ratio of Bax/Bcl-2 in DC2.4 cells treated with HMGB1. By contrast, SESN2 overexpression obviously alleviated HMGB1-induced cell death, evidenced by downregulating apoptotic rate and apoptotic-related proteins. Furthermore, in the in vivo experiments, the apoptosis was markedly elevated in sesn2^−/−^ DCs after CLP. These data further confirm the protective impact of SESN2 on apoptosis of DCs in the setting of sepsis relevant condition.

We further focused on the response of ERS in DCs to explore the precise mechanisms underlying cytoprotective actions of SESN2 against apoptosis induced by HMGB1 or during sepsis. ERS, a double-edged sword, plays a critical role in modulating the balance between homeostasis and apoptosis of cells^39,40^. It is vital in the pathological process of inflammation and sepsis^41^, and appears to be one of the lethal contributors to immune dysfunction and lymphocyte apoptosis^42^. It has been demonstrated that the upregulation of SESN2 is uniquely induced under ERS response^43^. Conversely, SESN2 exerts a marked influence on the response of ERS. In the current study, we noticed the co-location of SESN2 and ER, and SESN2 interacted with ATF4, indicating a close interaction between SESN2 with ERS response. Moreover, pretreatment with PERK inhibitor could obviously decrease the expression of SESN2 in DC2.4 cells induced by HMGB1, suggesting the significance of PERK signaling in regulation of SESN2 expression. It was reported that SESN2 protected against hepatocyte injury mediated by ERS, and related cell death was augmented in sesn2^−/−^ mice^20^. Thus, our data implicate the marked influence of SESN2 on ERS response in DCs exposed to acute inflammatory insults.

PERK-ATF4-CHOP pathway is one of the key mechanisms in ERS-related cell death^44,45^. CHOP is a necessary transcription factor for ERS-mediated cell demise in response to various pathological conditions^44,46^, and a critical signal to modulate the expression of Bcl-2 family member^45–48^. Herein, it was revealed that SESN2 inhibited the upregulation of CHOP expression induced by the prototypical ERS-mediated cell death in DC2.4 cells. HMGB1 stimulation resulted in significant activation of ERS signaling pathway, showing enhanced GRP78 expression and the activation of PERK, ATF4, as well as CHOP. Thus, the excessive ERS response under high concentration of HMGB1 contributed greatly to the apoptosis of DC2.4 cells, and SESN2 played an important role in protecting DCs against ERS-mediated apoptosis. In addition, it was found that the response of ERS and related cell death in DC2.4 cells under HMGB1 stimulation was markedly aggravated after SESN2 knockdown. The distinctly upregulation of CHOP supported the notion that SESN2-knowdown led to ERS out of balance and inclined to apoptotic response. On the contrary, overexpression of SESN2 significantly alleviated ERS response, restored ER homeostasis, and abated ERS-induced cell death, by the evidences in downregulating ERS sensor and ERS-related apoptotic proteins. More importantly, PERK signaling inhibitor weakened the protective effect of SESN2 on DC2.4 cells evoked by HMGB1. In addition, the extent of ERS as well as ER morphology was distinctly exacerbated in sesn2^−/−^ mice after sepsis, and sesn2^−/−^ knockout increased 24-h mortality in mice suffered from septic challenge. Taken together, SESN2 appears to be an important regulator in protection against apoptosis of DCs induced by inflammatory stimulation, which is associated with PERK-ATF4-CHOP signaling transduction.

In the present experiments, we demonstrated for the first time that the activation of SESN2 in apoptotic signaling after HMGB1-stimulated DCs played a protective role in DC2.4 cells by regulating the extent of ERS response. HMGB1 or TM stimulation was able to enhance the expression of SESN2 and the apoptotic rate of DC2.4 cells. SESN2-knockdown exacerbated HMGB1-induced cell apoptosis via aggravating ERS response and ER dilation or fragmentation. Conversely, SESN2 over-expression remarkably attenuated HMGB1 or TM-mediated cell apoptosis, and alleviated the morphologic alterations of ER and ERS response. Importantly, in a septic model of mice, knockout of SESN2 markedly aggravated the cell death of DCs mediated by ERS-related apoptosis. Thus, these results indicate that SESN2 appears to be a potential regulator to negatively modulate the apoptotic ERS response through inhibiting PERK-ATF4-CHOP pathway and exerts a protective impact on apoptosis of DCs following septic insults.

Nonetheless, our study suffers from some limitations. Firstly, we do not show direct evidence that ERS-mediated DC apoptosis by interfering ER-related molecules, thus we cannot conclude that ERS-induced pathway plays a decisive and ultimate role in HMGB1-induced apoptosis of DCs. Secondly, due to the limited number of sesn2^−/−^ mice, we only observed the survival rate of CLP mice at 24 h without recording the 7-day survival rate. Thirdly, it is deserve further study to elucidate the precise signaling pathways of ERS response by silencing ERS-related molecules in SESN2 transgenic mouse. Moreover, clinical studies should be followed to strengthen such interesting observation in the near future.

## Materials and methods

### Mice

Six-week-old male, C57BL/6J mice (weight range 20–25 g) were provided by the Institute of Laboratory Animal Science, Peking Union Medical College, Beijing, China. Sesn2^−/−^ mice in the C57BL/6J background were constructed by Shanghai Model Organisms Center, Shanghai, China. All mice were maintained in SPF conditions with a 12-h light/dark cycle. All experimental manipulations were conducted in accordance with the National Institutes of Health Guide for the Care and Use of Laboratory Animals, with the approval of the scientific Investigation Board of the Chinese PLA General Hospital, Beijing, China. WT and sesn2^−/−^ mice were randomly divided into two group, respectively: WT-sham group, WT-CLP group, sesn2^−/−^-sham group, sesn2^−/−^-CLP group, *n* = 6 per sham group, *n* = 11–13 per group.

### Reagents

DC2.4 cells (the murine DC cell line) were purchased from ATCC, Shanghai, China. CD11c^+^ (N418) MicroBeads were purchased from Miltenyi Biotec GmbH, Bergisch Gladbach, Germany. Recombinant HMGB1 was purchased from R&D System, Minneapolis, MI. TM was purchased from Sigma, St. Louis, MO. RPMI 1640, fetal calf serum, glutamine, penicillin, streptomycin, and HEPES were purchased from TianRunShanda Biotech Co. Ltd., Beijing, China. Annexin-V-PE and 7-AAD apoptosis detection kits were purchased from BD, San Diego, CA. Rabbit anti-mouse SESN2 (ab178518), rabbit anti-mouse GRP78 (ab21685), and rabbit anti-mouse ATF4 (ab184909) monoclonal antibodies were purchased from Abcam, Cambridge, MA. Rabbit anti-mouse caspase-3 (#9662), mouse anti-mouse cleaved-caspase-3 (#9661), mouse anti-mouse caspase-9 (#9508), rabbit anti-mouse Bcl-2 (#15071), rabbit anti-mouse Bax (#2772), rabbit anti-mouse PERK (#5683), rabbit anti-mouse p-PERK (#3179) monoclonal antibodies were purchased from Cell Signaling Technology, Danvers, MA. Mouse anti-mouse SESN2 (sc-393195) was purchased from Santa Cruz Biotechnology, Santa Cruz, CA. ER-tracker red was purchased from Invitrogen, California. Hoechst 33342 was purchased from R&D System, Minneapolis, MI. Fluorescein isothiocyante (FITC)-mouse anti-rabbit IgG was purchased from Santa Cruz Biotechnology, Santa Cruz, CA. Triton X-100 was purchased from Sigma, St. Louis, MO. CoraLite594- conjugated goat anti-mouse IgG (H + L) was purchased from Proteintech Group, Rosemont, IL. Pierce co-IP kit was purchased from Thermo Fisher Scientific, Waltham, MA. GSK2656157 was purchased from was purchased from Selleckchem, Houston, TX.

### Mouse model of sepsis induced by CLP

Polymicrobial sepsis was induced by CLP in mice. After anesthesia, the abdominal area was disinfected. A 1.0-cm incision was performed along the midline of the abdomen, and then the cecum was identified and exposed. A specified percentage of the cecum was ligated and punctured twice with a 21-gauge needle, and then the cecum was punctured to induce sepsis. Next, the cecum was returned to abdominal cavity. After surgery, 1 ml of 0.9% normal saline was injected subcutaneously. Mice exhibited lethargy, diarrhea and piloerection in the first 6 h after surgery, indicating the successful establishment of septic model. In the sham group, mice underwent the same surgical procedure except for the ligation and puncture step.

### Isolation of splenic DCs

Under aseptic condition, mice spleens were obtained and washed in twice with precooled phosphate-buffered saline (PBS). Mononuclear cells were isolated, and then splenic DCs were isolated from murine mononuclear cells using a CD11c^+^ dendritic cell isolation kit (Miltenyi Biotec, Bergisch Gladbach, Germany) with a positive selection MS column following manufacturer’s instructions. The selected DCs were obtained by centrifugation at 200 g for 10 min (min), and the supernatant was discarded. Then these isolated cells were cultured in Roswel Park Memorial Institute medium (RPMI 1640) with 10% heated-inactivated FBS, containing 100 U/ml penicillin and 100 μl/ml streptomycin at 5% CO_2_, 37 °C in a humidified incubator, or used for experiments.

### Cell culture and stimulation

DC2.4 cells were cultured in RPMI 1640 with 10% heated-inactivated FBS, containing 100 U/ml penicillin and 100 μl/ml streptomycin. Cells were cultured at 5% CO_2_, 37 °C in a humidified incubator. Before all experiments, cells were incubated for 24 h. DC2.4 cells were cultured with or without HMGB1 stimulation (cultured with 10 ng/ml HMGB1 for 8, 24, and 48 h, or cultured with HMGB1 for 48 h at different concentrations of 1, 10, and 100 ng/ml, respectively). After indicated stimulation, cells were collected for Western blot analysis and measurement by flow cytometry and laser scanning confocal microscope (LSCM, Leica, Mannheim, Germany), respectively.

### Total RNA extraction and reverse transcription-polymerase chain reaction analysis

After cells were stimulated as indicated above, total RNA from DC2.4 cells (2 × 10^6^) were extracted by using Trizol kits following manufacturer’s instruction. Primers for SESN2 were 5′-GCTGCTGGATGAGAAGTTCC-3′ (forward) and 5′-CCAAAGACGCAGTGGATGTA-3′ (reverse). Primers for β-actin, a housekeeping gene, were 5′-TGCGTGACATCAAAGAGAAG-3′ (forward) and 5′-TCCATACCCAAGAAGGAAGG-3′ (reverse). Thermal cycling conditions were 95 °C for 10 min, followed by 35 cycles of 95 °C for 40 s (s), 60 °C for 30 s, and 72 °C for 25 s, and a final extension period of 5 min at 72 °C.

### Western blot analysis and co-immunoprecipitation

Cells (3 × 10^6^) were collected and washed twice with ice-cold PBS, and lysed with lysis buffer (150 mM NaCl, 1.0% NP-40 or 0.1% TritonX-100, 0.5% sodium deoxycholate, 0.1% sodium dodecyl sulfate (SDS), 50 mM Tris-HCl pH 8.0, protease inhibitors, and phosphatase inhibitors). After incubation on ice for 30 min, the homogenate was centrifuged at 14,000 rpm for 15 min at 4 °C, and then boiled at 95 °C for 5 min after mixing with SDS-loading buffer. The samples were separated with 8–12% SDS polyacrylamide gel electrophoresis (Pulilai Co., Beijing, China), and then transferred to nitrocellulose membranes and blocked with 10% milk in TBST at room temperature for 2 h. Specific antibodies were used to determine expressions of SESN2, GRP78, CHOP, p-PERK, PERK, ATF4, caspase-3, cleaved-caspase-3, caspase-9, Bcl-2, and Bax, respectively. A monoclonal anti-β-actin antibody was used as a control for protein loading. Immunoreactivity was visualized by ECL detection system (Amersham Biosciences, Uppsala, Sweden). Protein levels were quantified by densitometric analysis. Co-IP was done according to the manufacturer’s protocol. SESN2 antibody was immobilized for 120 min using AminoLink Plus coupling resin. Then resin was washed and incubated with total cell lysate overnight at 4 °C. After incubation, resin was washed twice and protein eluted by elution buffer. A negative control using control agarose resin to assess nonspecific binding received the same treatment as the co-IP samples. Samples were analyzed by Western blot analysis.

### Laser scanning confocal microscopy

Morphological alterations of ER in DC2.4 cells and splenic DCs were performed by LCSM. The ER-tracker probe was used to selectively stain the ER of live cells. After incubating the cells for approximately 15–30 min at 37 °C, cells were collected and washed with PBS for three times, and fixed with 4% paraformaldehyde for 20 min, then permeabilized with 0.1% Triton X-100 for 20 min at room temperature. Thereafter, cells were pre-blocked with 1% bovine serum albumin in distilled water for 30 min and stained with anti-SESN2 antibody (1:200) or anti-ATF4 antibody (1:200) or anti-GRP78 antibody (1:100) overnight at 4 °C. By washed with PBS for three times, cells were stained with second antibody (FITC-goat-anti-IgG, PE-goat-anti-IgG) for 1 h at 37 °C followed by PBS washes for three times. After being washed, the nuclei were stained with 4′, 6-diamidino-2 -phenylindole (DAPI). The cells were observed using a laser scanning confocal microscope (Leica, Mannheim, Germany).

### Flow cytometric analysis

The apoptotic rates of DCs were determined using simultaneous Annexin-V-PE and 7-AAD, and the negative control was designed. Cells (5 × 10^5^) were cultured in 96-well flat bottom plates. After treatment with or without HMGB1, cells were collected and washed three times with cold PBS, and then resuspended in 100 μl binding buffer, to which added 5 μl Annexin-V-PE and 5 μl 7-AAD. Incubation in darkness for 15 min at room temperature, the cells were diluted with 200 μl binding buffer and analyzed by flow cytometry within 1 h using a FACScan (BD Biosciences, Mountain View, CA).

### Hoechst 33342 analysis

After stimulation with HMGB1 (100 ng/ml for 48 h), morphological changes in apoptotic DC2.4 cells were measured by nuclear staining with the bisbenzimidazole dye Hoechst 33342 solution. Cells were exposed to 5–10 μg/ml Hoechst 33342 solution at 37 °C for 30–60 min. Then Hoechst 33342 solution was removed from cells, and fluorescence microscopy was used to evaluate the apoptotic cells with image representative fields for each experiment.

### SESN2 RNA lentivirus generation and transfectio**n**

Over-expression RNA to SESN2 (SESN2-LV-RNA) or small interference RNA to SESN2 (SESN2-siRNA) was synthesized by Hanbio Biotechnology Co., Shanghai, China. For over-expression or knockdown of SESN2 expression, DC2.4 cells were introduced with recombinant lentiviruses that carried the SESN2-LV-RNA or SESN2-siRNA. The recombinant lentiviruses were transduced according to the manufacture’s instruction. The transduction efficiency for DC2.4 cells in vitro was >80%. After transfection for 3 days, the efficiency of overexpression or knockdown was checked by Western blotting for SESN2 expression.

### Statistical analysis

All results were represented as mean ± standard deviation of more than three independent experiments. One-way analysis of variance (ANOVA) was used to analyze significant differences among the groups, and student *t* test was needed for assessing significant differences of intergroup. *P* values less than 0.05 were considered statistically significant.

## Supplementary information

author contribution
